# Surface-enhanced Raman Spectroscopy in urinalysis of hypertension patients with kidney disease

**DOI:** 10.1038/s41598-024-53679-9

**Published:** 2024-02-06

**Authors:** Alberto C. Espinosa-Garavito, Elkin Navarro Quiroz, Nataly J. Galán-Freyle, Gustavo Aroca-Martinez, Samuel P. Hernández-Rivera, Joe Villa-Medina, Maximiliano Méndez-López, Lorena Gomez-Escorcia, Antonio Acosta-Hoyos, Lisandro Pacheco-Lugo, Fabián Espitia-Almeida, Leonardo C. Pacheco-Londoño

**Affiliations:** 1 Centro de Investigaciones en Ciencias de la Vida, Facultad de Ciencias Básicas y Biomédicas, Universidad Simón Bolívar, 080002 Barranquilla, Atlántico Colombia; 2 Clínica de la Costa, 080020 Barranquilla, Atlántico Colombia; 3 Center for Chemical Sensors, DHS SENTRY COE, University of Puerto Rico-Mayaguez, Mayaguez, PR 00681 USA; 4 Center of Pharmaceutical Research, Procaps Laboratories, 080002 Barranquilla, Colombia; 5 Grupo de Química y Biología, Departamento de Química y Biología, Universidad del Norte, Km 5 Vía Puerto Colombia, 080001 Barranquilla, Colombia

**Keywords:** Biotechnology, Molecular biology, Biomarkers, Health care, Molecular medicine, Nephrology, Nanoscience and technology

## Abstract

Arterial hypertension (AH) is a multifactorial and asymptomatic disease that affects vital organs such as the kidneys and heart. Considering its prevalence and the associated severe health repercussions, hypertension has become a disease of great relevance for public health across the globe. Conventionally, the classification of an individual as hypertensive or non-hypertensive is conducted through ambulatory blood pressure monitoring over a 24-h period. Although this method provides a reliable diagnosis, it has notable limitations, such as additional costs, intolerance experienced by some patients, and interferences derived from physical activities. Moreover, some patients with significant renal impairment may not present proteinuria. Accordingly, alternative methodologies are applied for the classification of individuals as hypertensive or non-hypertensive, such as the detection of metabolites in urine samples through liquid chromatography or mass spectrometry. However, the high cost of these techniques limits their applicability for clinical use. Consequently, an alternative methodology was developed for the detection of molecular patterns in urine collected from hypertension patients. This study generated a direct discrimination model for hypertensive and non-hypertensive individuals through the amplification of Raman signals in urine samples based on gold nanoparticles and supported by chemometric techniques such as partial least squares-discriminant analysis (PLS-DA). Specifically, 162 patient urine samples were used to create a PLS-DA model. These samples included 87 urine samples from patients diagnosed with hypertension and 75 samples from non-hypertensive volunteers. In the AH group, 35 patients were diagnosed with kidney damage and were further classified into a subgroup termed (RAH). The PLS-DA model with 4 latent variables (LV) was used to classify the hypertensive patients with external validation prediction (P) sensitivity of 86.4%, P specificity of 77.8%, and P accuracy of 82.5%. This study demonstrates the ability of surface-enhanced Raman spectroscopy to differentiate between hypertensive and non-hypertensive patients through urine samples, representing a significant advance in the detection and management of AH. Additionally, the same model was then used to discriminate only patients diagnosed with renal damage and controls with a P sensitivity of 100%, P specificity of 77.8%, and P accuracy of 82.5%.

## Introduction

Arterial hypertension (AH) is defined as persistently elevated blood pressure, such as systolic blood pressure of ≥ 130 mmHg and/or diastolic blood pressure (DBP) of ≥ 90 mmHg, taken as an average of three correctly measured readings^1–3^. Furthermore, AH has been closely associated with different alterations, considering the intrinsic systemic nature of blood circulation, which frequently affects the kidney to lead to the development of kidney diseases (KDs)^4^ that encompass a great heterogeneity of pathophysiological processes that often coexist and overlap with others. Hypertension and KD are interrelated, and both can be the cause and/or consequence of each other^5^.

AH can induce kidney damage through damage to the blood vessels, sodium dysregulation, increased sympathetic nervous system, scar formation, and hardening of the glomeruli. At the cellular level, hypertension can induce significant metabolic changes that contribute to these pathological effects. These changes may involve increased production of reactive oxygen species, the activation of the renin–angiotensin–aldosterone system, and the alteration of the levels of various metabolites. These metabolic changes can trigger the accumulation of toxic waste products and the dysfunction of kidney cells^6^. In several cases, most people with AH do not present signs or symptoms^7^, thereby making it a silent disease. AH is a global public health concern because the number of cases worldwide has increased from 650 million to 1,280 million in the last 30 years^8^, and approximately one-third of adults are estimated to have hypertension^2^. Moreover, as per the World Health Organization, an alarming 46% of hypertensive adults remain unaware of their condition^7^, and tragically, 8.5 million deaths have been attributed to the association of hypertension with other comorbidities^8^.

Although the AH diagnosis is simple (it is made through conventional blood pressure measurements in a medical office or through ambulatory blood pressure monitoring), the devices for such measurements must be validated according to standardized conditions and protocols. In addition, the possible masking effect due to the white-coat effect, the discomfort in some patients due to the follow-up time, and the limited availability of access to health systems make it challenging to identify hypertension in certain patients. Therefore, technological tools that can facilitate the classification of hypertensive patients through urinalysis in populations at a higher risk of developing kidney damage due to hypertension can contribute to the clinical characterization of the population to support decision-making. This study developed a methodology for classifying hypertensive patients by analyzing urine samples using surface-enhanced Raman spectroscopy (SERS) coupled with gold nanoparticles (AuNPs) and supported with multivariate statistical analysis (chemometrics).

Raman spectroscopy is an optical spectroscopic technique based on inelastic scattering, which is defined by the shifting energy. This shift is calculated by the difference between the energy of incident photons and the energy of emitted photons, which is equivalent to the vibrational mode energy of the interrogated molecules^4^. Raman spectroscopy offers the advantages of minimal treatment of samples, minimal interference by water molecules, and nondestructive and automatable sample analysis, among others^9^. Another advantage of using Raman for this assay is the possibility of finding spectral markers (functional groups) associated with hypertension patients, which could aid in elucidating the mechanism by which hypertension affects the urine chemical environment. For this reason, the direct analysis of urine samples is of great interest. Nonetheless, the Raman phenomenon suffers from a disadvantage in that its occurrence probability is weaker than that of other techniques, such as infrared spectroscopy. However, this limitation can be overcome by employing SERS. The implementation of AuNPs is useful owing to their plasmonic properties that enhance the Raman signal on the order of 10^4^–10^9^ times, thereby providing greater sensitivity. In addition, multivariate statistical analysis provides evidence of pattern differences in the spectral signals from complex matrices such as urine samples.

The applications of Raman spectroscopy have attracted interest as a promising alternative technique to address certain disadvantages in diagnosing pathologies^9^. For example, SERS methodologies have been used for the detection of different types of cancer^10–12^ from urine samples, such as breast cancer^13^, prostate cancer^14^, and colorectal cancer^15^, and conventional Raman spectroscopy has been used to detect cervical cancer^16^. Other studies have used Raman spectroscopy for the diagnosis of kidney damage by evaluating metabolites from urine samples, such as albumin for diabetic patients^17^; creatinine, urea, and glucose for hypertensive diabetic patients^18^; hydroxybutyrate, alanine, creatinine, and porphyrins for the diagnosis of renal failure^19^; and SERS analysis to determine proteinuria from urine samples^20^. In particular, SERS with silver nanoparticles has been used to determine hypertension in blood samples. It evaluates the changes in erythrocytes generated by hypertension^21^ The biochemical analysis of urine samples is performed for the classification and diagnosis of healthy people and those with diabetes and hypertension by using Raman spectroscopy^22^, and the noninvasive and prospective diagnosis of coronary heart disease from urine samples is performed by using SERS based on the detection of platelet-derived growth factor-BB^23^.

In this study, we have proposed a direct model for the identification of hypertensive patients through enhanced Raman signals in urine samples coupled with AuNPs, supported by the chemometrics technique Partial Least Squares-Discriminant Analysis (PLS-DA). This alternative methodology for molecular pattern detection in urine samples associated with hypertension allows the distinguishing of hypertensive from nonhypertensive patients based on the spectral patterns obtained from the urine samples. The PLS-DA models were validated and evaluated for their sensitivity, specificity, and accuracy, among other variables.

## Materials and methods

### Materials

Hydrogen tetrachloroaurate (III) trihydrate (HAuCl_4_·3H_2_O) ≥ 99.9%, Sigma‒Aldrich (St Louis, MO USA); sodium citrate dihydrate, granular (C_6_H_5_Na_3_O_7_·2H_2_O) 99.3%, J. T. Baker–Fisher Scientific (Edo. de Mex. Mexico), MiliQ water Arium Comfortm, Sartorius AG (Göttingen, Germany); CLARIO Starplus spectrophotometer (BMG-LABTECH The Microplate Reader Company, Ortenberg Germany); Zetasizer LAB (Malvern Panalytical a spectris company, United Kingdom). Raman 785 L, Wasatch Photonics (Orlando, FL, USA).

### Patients, sample collection, and storage

The Ethics Committee of Universidad Simón Bolívar, Barranquilla–Colombia, approved this study. In addition, written informed consent was obtained for the collection of urine samples from all study subjects (approximately 30 mL for each patient). A total of 162 urine samples were collected from volunteer patients who attended the clinic (Clinica de la Costa, Barranquilla–Colombia), including 87 AH patients and 75 healthy volunteers. The collected urine samples were identified and labeled AH_N° patients for those with a diagnosis of AH and HV_N° patients for healthy volunteers. Then, the urine samples were separated in aliquots of 500 µL and stored at − 80 °C until further analysis. In the AH group, 35 patients were diagnosed with kidney damage and were further classified into a subgroup termed (RAH). Of the 162 urine samples, 122 were used to generate the prediction model, and 40 were used for external validation. The separation of the samples for the training (to generate the prediction) and external validation of the model was performed randomly, assigning 75% for training and 25% for the validation of the model.

### Synthesis and characterization of AuNPs

AuNPs were synthesized following the protocol described by Hermanson et al.^24^, albeit with some modifications. AuNPs were prepared via chemical reduction of 20 mM HAuCl_4_(III) solution using sodium citrate 2% (w/v). Briefly, 1250 µL of 20 mM HAuCl_4_ was added to 100 mL of boiling Type 1 water under constant stirring at 400 rpm. Then, 500 µL of sodium citrate 2% (w/v) was added to the solution and stirred continuously for 30 min. The color change of the solution indicated the formation of monodisperse colloidal gold particles. The AuNPs were further characterized by determining their absorption maximum with a UV–Vis scan at 350–800 nm (CLARIO Starplus) and by determining the hydrodynamic radius of the colloidal solution through dynamic light scattering (Zetasizer)^25–28^.

### Urine sample preparation and SERS measurements

The urine sample aliquots were unfrozen for approximately 30 min at room temperature, and the individual urine samples were homogenized before acquiring their Raman spectra. For the SERS measurements, 150 µL of urine was mixed with 150 µL of AuNP solution. Then, 200 µL of the mixture (urine-AuNPs) was centrifuged at 2000 rpm for 5 min to generate a pellet at the bottom of the centrifuge tube. The generated pellet was dissolved in 50 µL of Type 1 water, and a drop of 5 µL of the urine-AuNP mixture was deposited on a copper plate, after which the Raman spectra were measured by focusing a 785 nm laser inside the drop. The integration time was 1 s, with a laser power of 100 mW. Five Raman spectra were also acquired and averaged in order to have one spectrum for each sample.

### PLS-DA model

Chemometric analysis was performed by using MATLAB® 8.6.0.267246 (R2015b; Math Works Inc. Natick, USA) and PLS Toolbox 8.1 (Eigenvector Research, Inc., Wenatchee, WA, USA). PLS-DA is a classification technique derived from the Partial Least Squares (PLS) algorithm. It identifies latent variables that best separate classes in a multivariate space. The PLS-DA algorithm takes the original predictor variables and transforms them into a new set of variables, known as latent variables. These latent variables are mathematical constructs derived from the original variables, designed to capture the maximum amount of relevant information regarding the observed variation in the response variable's classes. This method is particularly useful for datasets with many, possibly correlated, predictor variables and for situations where the predictors outnumber the observations. In the evaluation of the performance of predictive models such as PLS-DA, accuracy, sensitivity and specificity are key metrics. Accuracy reflects the overall correctness of the model, indicating the proportion of correct predictions. Sensitivity, or the true positive rate, measures the model's ability to correctly identify actual positives, which is crucial for not missing cases in scenarios like disease screening. Specificity, or the true negative rate, assesses the model's capacity to dismiss non-cases, preventing false alarms correctly.

In this study, the spectral data of different samples were preprocessed using different preprocessing such as standard vector normalization (SNV), multiplicative scattering correction (MSC), derivative, and combinations of the derivative and the priors. the best result was the 2nd derivative (2ndD; order: 2; window: 15 pt; tails: polyinterp)^29^, which effectively highlights the maximums and minima of the spectral variables within the Raman spectra of urine samples from both healthy subjects and hypertensive patients, additionally 2nd derivative is for correcting baseline distortions in the Raman spectrum.

These models were evaluated through the parameters of the confusion matrix, such as sensitivity, specificity, and precision for cross-validation (CV), and prediction of sample by external validation (P)^30^. In addition, the area under the receiver–operator curve (ROC), which is a probability curve that displays the performance of a classification model, was applied. A ROC curve is a graphical plot that illustrates the diagnostic ability of a binary classifier system as its discrimination threshold is varied. It is created by plotting the true positive rate (sensitivity) against the false positive rate (1 − specificity) at various threshold settings. The area under the ROC curve (AUC) is a measure of the model's ability to correctly classify the outcomes. The closer the curve follows the left-hand border and then the top border of the ROC space, the more accurate the test. Conversely, a curve that lies close to the diagonal represents a random guess. Furthermore, an analysis of variable importance in projection (VIP) was executed to identify the spectral variables contributing significantly to the discrimination between classes. A higher VIP score suggests that a variable is important for the model, and these scores are often used to select features during the process of model optimization. Variables with VIP scores greater than 1 are typically considered significant.

### Compliance with guidelines and regulations

We would like to confirm that all procedures and methodologies used in this study were carried out in strict compliance with the relevant guidelines and regulations as stipulated by the journal's editorial policy. Additionally, this study has been approved by the Simon Bolivar University ethics committee (PRO-CEI-USB-0425-00), ensuring the integrity and ethics of our research. We have taken all necessary precautions to ensure that the methods employed are consistent with established standards and to guarantee the validity and reproducibility of our results. We have obtained all required permissions and have ensured that our methods are transparent, ethical, and rigorous.

## Results

### Synthesis and characterization of AuNPs

**Figure 1:**
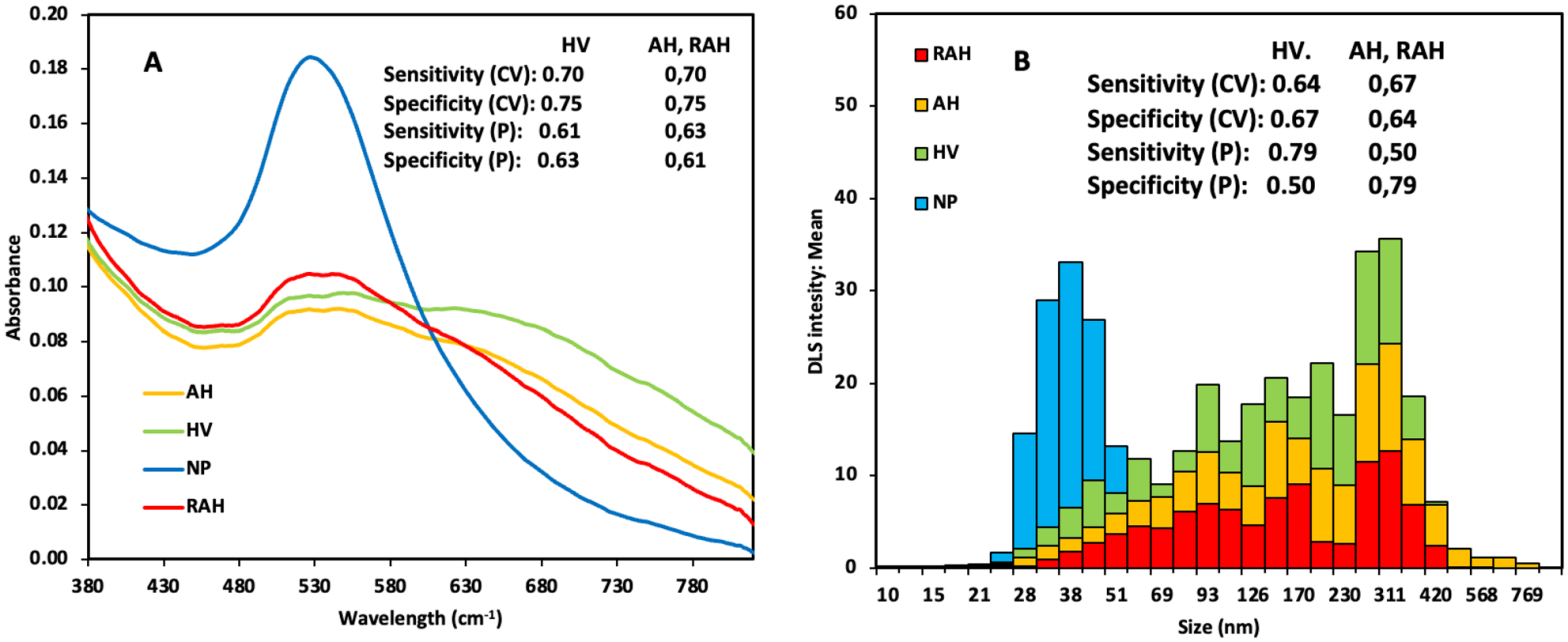
Synthesis and characterization of AuNPs: (**A**) Maximum adsorption of AuNP solution (blue line, NP), urine AH patients with AuNP solution (yellow line, AH), urine HV patients with AuNP solution (green line, HV) and urine HV patients diagnosed with kidney damage RAH (red line, RAH). (**B**) Particle size distribution of AuNPs solution polygon color blue, urine AH patients with AuNPs polygon color yellow, urine HV patients with AuNPs polygon color green and urine RAH patients with AuNPs polygon color red. Tables inserted in the figures: sensitivity and specificity for cross-validation (CV) and external validation (P).

AuNPs were synthesized as per the protocol of Hermanson et al*.* (2013), albeit with some modifications. A color change of the suspension from light yellow to dark blue to red was observed for monodisperse colloidal AuNPs. The optical property (plasmon resonance) of the AuNP solution was verified with a maximum absorption at 530 nm, corresponding to AuNPs approximately < 50 nm in size (Fig. 1A). In addition, the AuNP particle size distribution was measured by dynamic light scattering (DLS) using Zetasizer LAB equipment. The AuNP particle size distribution was 37 ± 1 nm (Fig. 1B).

### PLS-DA models

Three PLS-DA models were generated; the first PLS-DA model was created with the absorbance data of the visible spectrum of AuNPs. Figure 1A displays the visible spectrum of AuNPs (blue line, NP), urine AH patients with AuNP solution (yellow line, AH), urine HV patients with AuNP solution (green line, HV) and urine RAH patients with AuNP solution (red line, RAH). Figure 1A shows that when the AuNPs are mixed with urine, a new absorption band appears, which may be attributed to the interaction of the AuNPs with the existing metabolites in urine, possibly due to an agglomeration generated in the sample. The sensitivity and specificity for cross-validation (CV) and external validation (P) for this model were 0.70, 0.75, 0,63, and 0,61, respectively (see table inserted in Fig. 1A); these values indicate acceptable discrimination, suggesting that the concentrations and the type of metabolites in urine differ between HV patients and AH patients. A second model PLS-DA, based on the size distribution data, was obtained by DLS, as verified by the size of the AuNP clusters with urinary metabolites measured by DLS (see Fig. 1B), giving a sensitivity and specificity of 0.67, 0,64, 0,50, and 0,79, respectively (see table inserted in Fig. 1B).

A third PLS-DA model was generated using the second derivative of SERS spectral data of urine samples from HV and AH patients. The root-mean-standard error for cross-validation (RMSECV) of the model to different latent variables was 0.413, 0.357, 0.299, 0.237, 0.261, and 0.285 and the lowest error value was with an error of 0.237 with 4 latent variables. The root means square error of calibration (RMSEC) of 0.2269. The model’s performance was evaluated through the parameters of the confusion matrix, such as sensitivity and specificity. Sensitivities and specificities were calculated for the cross-validation (CV), and validation external (P) of prediction models^31^. The results indicated a sensitivity of CV of 0.769 (76.9%), sensitivity P of 0.864 (86.4%), specificity of CV of 0.772 (77.2%), specificity of P of 0.778 (77.8%), accuracy of CV of 0.770 (77.0%), and accuracy P of 0.825 (82.5%) (see Table 1). The same model was then used to discriminate only patients diagnosed with renal damage and controls, and a new confusion matrix was generated; the sensitivity and specificity were found to be better than for discrimination considering hypertension (see Table 1). The results for RAH have a sensitivity of CV of 0.843 (84.3%), sensitivity P of 1.000 (100.0%), specificity of CV of 0.772 (77.2%), specificity of P of 0.778 (77.8%), accuracy of CV of 0.795 (79.5%), and accuracy P of 0.825 (82.5%).

**Table 1:** PLS-DA classification confusion matrix for hypertensive models.

Hypertensive model					
	AH, RAH	HV	Sensitivity	Specificity	Accuracy
Confusion table (cross validation)					
Predicted as AH, RAH	50	13	0.769	0.772	0.770
Predicted as HV	15	44			
Confusion table (prediction)					
Predicted as AH, RAH	19	4	0.864	0.778	0.825
Predicted as HV	3	14			

Hypertensive diagnosis with kidney damage model					
	RAH	HV	Sensitivity	specificity	Accuracy
Confusion table (cross validation)					
Predicted as RAH	22	13	0.846	0.772	0.795
Predicted as HV	4	44			
Confusion table (prediction)					
Predicted as RAH	9	4	1.000	0.778	0.852
Predicted as HV	0	14			

**Figure 2:**
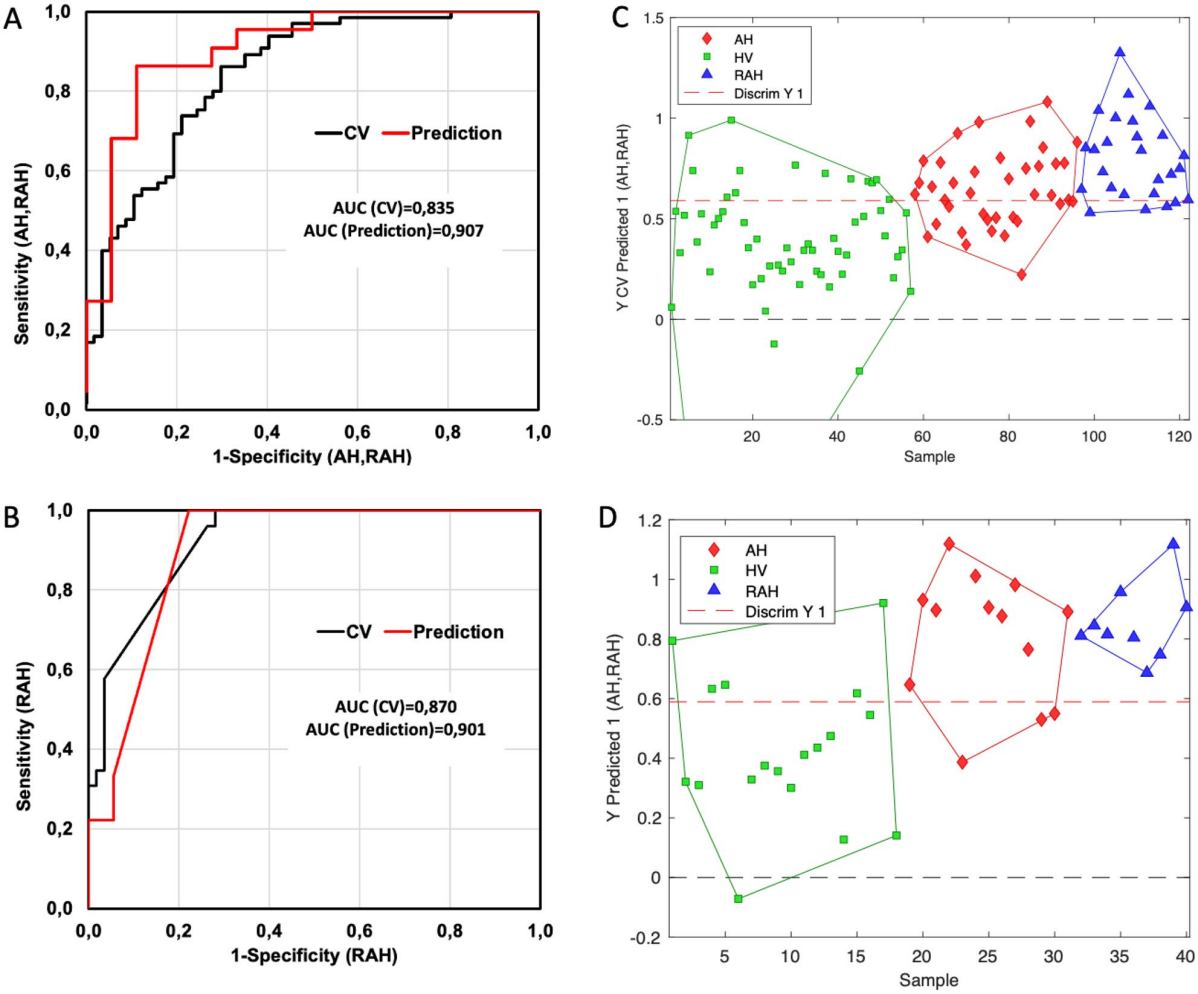
(**A**) The ROC curve of the hypertensive model, (**B**) ROC curve of the hypertensive diagnosed with kidney damage model: cross-validation (black line) and prediction (red line). (**C**) Classification of patients according to Y predicted the cross-validation (**D**) Classification of patients according to Y predicted the external validation, the green points for HV, the red points for AH, and the blue points for RAH.

In addition, the ROC curve of the third PLS-DA model was generated to verify how strong the contribution of the spectral variables was with respect to the classification of HV, AH, and RAH patients. The ROC curve was evaluated with the specificity and sensitivity parameters for the CV and P model for AH + RAH (see Fig. 2A), and RAH (see Fig. 2B), with an AUC for CV of 0.835 (83.5%) and 0.870 (87.0%), respectively; and for P is 0.907 (90.7%) and 0.90.1 (90.1%), respectively. Figure 2C,D show the value predicted for AH in the classification of patients into two classes, HV and AH (with the RAH subclass), of the model classification for hypertension. In addition, the contribution of LV scores in the classification of patients displays two well-defined classes of HV and AH patients using a graph of scores with only the first 4 LVs. Notably, even with the obtained sensitivity and specificity, it was necessary to conduct validation to evaluate the clinical efficacy of the classification model for hypertensive patients.

Several machine learning methods were also tested, such as Logistic Regression, SVM, Random Forest, KNN, Decision Tree, Gradient Boosting, MLP, AdaBoost, Gaussian Naive Bayes, and Ridge Classification. We found that there was no significant improvement in the results. (see Supplementary Material).

**Figure 3:**
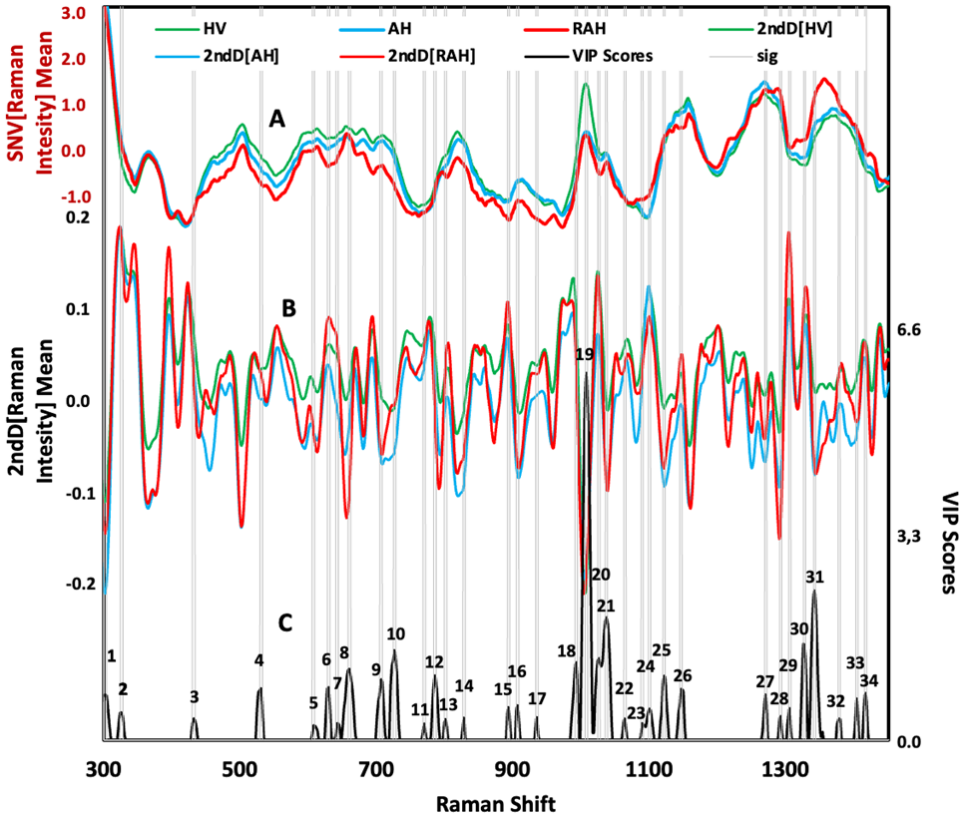
Relationship of Raman spectra with preprocessing and representative spectral bands: (**A**) Average data spectrum Raman (Baseline and Standard Normal Variate Scaling (SNV)) from AH (solid orange line) and HV (solid blue line). (**B**) Average of second-preprocessing data spectrum Raman from AH (solid orange line) and HV (solid blue line). (**C**) VIP scores > 1 of the Raman spectra matrix of urine samples-AuNPs (solid blue line).

To identify the significant spectral bands for the model, the VIP scores > 1 were identified and compared with the average Raman spectra and the signals generated by the [2ndD] preprocess (Fig. 3). A comparison of the spectrum (data) average of the urine samples from HV with AH was generated (Fig. 3A). Considerable variations were observed in the presence and intensity of spectral peaks in different regions between 400–570, 600–800, 900–1170, and 1200–1400 cm^−1^. When performing second-derivative preprocessing, as shown in Fig. 3B, the different spectral bands were highlighted, with the observation of the highest intensities in the spectral ranges 600–800, 970–1050, and 1300–1350 cm^−1^.

VIP is the measure that indicates the variables that are important and displays their contributions to the model. It helps to select the variables that will be used to develop the predictive model. The calculation of the VIPs of the variables is conducted by means of the weighted sum of the squared correlations between the components and the original variable and whose weightings correspond to the percentage variation explained by the PLS-DA component in the model^32,33^.

The VIP scores help identify the most important spectral regions that contribute to the optimal performance of the model^33^.

In the VIP scores of the Raman spectra matrix of urine samples for the classification model of hypertensive patients, 36 bands were identified that exceeded the limit value, some contributing to the classification model with more weight than others. A total of 36 Raman spectral bands that contribute to the PLS-DA classification model were assigned tentatively, according to the literature, and the bibliographic references of the main Raman bands in urine samples from HV and AH (Table 2).

**Table 2:** VIP scores matrix spectra Raman urine samples. n.r., not resolvable.

Signal	Value VIP scores	Urine peaks (cm^−1^)	Possible assignment
1	1.68	302	n.r
2	1.41	326	n.r
3	1.32	432	n.r
4	1.77	531	S–S stretching protein^14^
5	1.21	607	Creatinine, glycerol^34,35^
6	1.79	630	Glycerol, C–S gauche amino acid methionine^35^
7	1.25	642	Uric acid, C–C twisting mode of tyrosine^15,35^
8	**2.07**	**660**	**C–S stretching mode of cystine collagen type II**^17,35^
9	1.91	706	N–H Uric acid, C–S trans amino acid methionine^16,35^
10	**2.35**	**726**	**DNA/RNA bases, hypoxanthine, C–S protein, CH2 rocking adenine**^15,35^
11	1.24	770	Alanina^19^
12	1.97	786	Ring vibration cytosine, DNA O–P–O uracil, thymine^16,35^
13	1.31	801	Backbone geometry and phosphate ion interaction^35^
14	1.33	828	Glutathione, tyrosine PO_2_ stretch DNA phosphodiester, O–P–O stretching DNA/RNA^15,35^
15	1.49	894	Phosphodiester deoxyribose^35^
16	1.52	907	Creatinine, creatine, hydroxibutyrate^34^
17	1.34	935	C–C stretching mode of proline and valine and protein backbone^34^
18	**2.16**	**993**	**Ring vibration uric acid, phenylalanine**^16^
19	**6.53**	**1008**	**N–C–N stretching urea, phenylalanine**^34,35^
20	**2.23**	**1026**	**C–H stretching phenylalanine**^14^
21	**2.85**	**1037**	**n.r**
22	1.32	1064	Skeletal C–C stretch of lipids^35^
23	1.25	1089	Po2 stretch, phosphate, histidine, nucleic acid^15,16,35,36^
24	1.47	1100	C–C vibration mode of the gauche-bonded chain, amide III^35^
25	1.97	1121	C–N stretch protein backbone, vibrations C–O, C–C, C–N uric acid^16,37^
26	1.76	1146	C–C lipids, fatty acid^35,36^
27	1.68	1271	Amide III band in protein, amide III C–N stretch, C=C fatty acid, typical phospholipids^35^
28	1.35	1292	Interring stretching, cytosine^35^
29	1.47	1306	CH3/CH2 twisting or bending mode of lipid/collegen^35^
30	**2.40**	**1327**	**CH3CH2 wagging mode in purine bases of nucleic acids**^35^
31	**3.25**	**1342**	**CH3, CH2 twisting nucleic acid, wagging protein, G(DNA/RNA), CH deformation protein and carbohydrates**^14,35,37^
32	1.32	1378	Ring breathing modes DNA, paraffin^16,35^
33	1.62	1404	CH deformation^35^
34	1.71	1417	C=C stretching in quinoid ring^35^
35	1.17	1428	CH2 creatinine, valine^15,17^
36	1.20	1479	Amide III^35^

Significant values are in bold.

Examining the 36 spectral bands that exceeded the VIP score threshold, the analysis showed that 19 bands had VIP scores between 1 and 2, indicating a moderate impact on the model. Additionally, 8 bands registered more pronounced VIP scores > 2.0, specifically at Raman shift 660, 726, 993, 1008, 1026, 1037, 1327, and 1342 cm^−1^. These notable bands correspond to functional groups associated with essential biomolecules such as DNA, RNA, amino acids, proteins, carbohydrates, and uric acid, as confirmed by scientific literature (see Table 2). The differences in the levels and types of these metabolites between classes suggest metabolic changes that may be associated with an inflammatory response characteristic of hypertension, with subsequent effects on kidney function and possibly other organs. The analysis underscores the pertinence of urinary DNA, which encompasses DNA fragments shed from damaged or necrotic cells. In hypertensive conditions, elevated blood pressure can inflict mechanical and metabolic stress on renal tissues, instigating cellular demise and the subsequent liberation of DNA into the urine^38^. The detection of urinary non-coding RNAs (ncRNAs) and changes in amino acid and protein profiles reveal the complex interactions within the body's structural and functional systems, particularly in the milieu of hypertension and its renal manifestations. These findings highlight how an alteration in a specific organ, such as the kidney in hypertension, can arise from multiple causes and manifest in heterogeneous effects both locally and systemically, as exemplified by conditions such as acute kidney injury (AKI). Furthermore, this research reveals the intricate interrelationship between the genome and epigenome and how these genetic and epigenetic influences are reflected in phenotypic characteristics, having significant implications for organ health^39–41^. Additionally, the presence of urinary uric acid can provide information on the balance between uric acid production and excretion. In hypertension, elevated uric acid levels in the urine may reflect an overproduction or decreased renal excretion of uric acid^42^.

## Discussion

Hypertension is a silent disease that is associated with other comorbidities, accounting for 8.5 million deaths worldwide^8^, with a national prevalence in Colombia of 9.08% per 100 inhabitants^43^. The effect of hypertension on the clinical implications of kidney disease and chronic diseases makes it critical to search for tools that allow early and easy classification of patients with high blood pressure to prevent potential hypertensive kidney damage. This study aimed to develop a noninvasive, rapid, and sensitive method for the diagnosis of AH based on urine analysis using AuNPs and Raman spectroscopy. Enhanced Raman spectroscopy can be used to classify hypertensive and nonhypertensive patients quickly, simply, and economically. We hypothesized that the urine of hypertensive patients presents biochemical changes that can be detected by using AuNPs as Raman signal enhancement agents and by using PLS-DA as a multivariate classification method.

This study is relevant in its endeavor to develop alternatives to the conventional technique of blood pressure measurement for the diagnosis of a condition due to AH, which is a chronic disease that affects millions of people across the world and involves a high risk of developing cardiovascular, renal, and cerebrovascular diseases. In this study, we innovated the combined use of AuNPs and Raman spectroscopy as a photonic technique that facilitates the provision of chemical and structural information from biological samples without any prior preparation or the use of chemical reagents. AuNPs act as Raman signal enhancement agents by inducing an effect known as SERS, which increases the intensity of the spectrum by up to five orders of magnitude, thereby allowing detection of the molecules even at trace levels.

Our study used enhanced Raman spectroscopy to distinguish healthy patients from hypertensive patients and to detect the differences between the molecular composition of the urine of these patients. Moreover, the Raman spectra and PLS-DA allowed the classification of urine samples of the two classes under the study owing to the exclusive spectral characteristics of these samples that facilitated molecular assignments possible to the spectroscopic signals (Table 2). As seen in this table, several bands displayed highly significant scores that contributed significantly to the model, without leaving aside the bands that presented lower scores as they could be significant. This analysis was not based on a single factor or molecular pattern but rather on a combination of several factors, and the relevant information could be extracted from the Raman spectra through PLS-DA, which makes it possible to demonstrate the difference between HV and AH urine spectra and attribute the variations in the molecular patterns.

In the application of the PLS-DA model to classify hypertensive patients and healthy subjects, it was found that in a group of patients who were detected positive for hypertension with the conventional diagnostic tests, 86.4% gave positive results with this model, and in the group of patients who gave negative results with the conventional technique, 77.8% were deemed negative with the PLS-DA model. The present results for both specificity and sensitivity displayed values suitable as a proof of concept that promotes further larger-scale studies toward developing more robust models that guarantee accurate diagnosis. In addition, the present PLS-DA model could identify hypertensive patients with kidney damage, which propels the investigation of a new line of study. It is important to identify hypertensive patients with kidney damage, as they have a higher risk of progressing to chronic kidney failure or developing cardiovascular complications, which may necessitate renal replacement therapy.

In addition, in a clinical setting, the proposed method can be applied for the management of hypertensive patients with kidney damage, such as the possibility of adjusting antihypertensive treatment by preventing or delaying the progression of kidney damage, monitoring response to treatment, or evaluating long-term prognosis. In the future, the proposed method should be optimized, such as with the use of other urinary biomarkers that may be related to hypertensive kidney damage, the use of other metal nanoparticles that may have a different SERS effect, or the use of other statistical techniques that may optimize the classification model.

### Supplementary Information

Supplementary Information 1.

Supplementary Information 2.

## Data Availability

All data sets generated for this study are included in the manuscript/supplementary files.

## Abbreviations

AH: Hypertensive
HV: Controls without a diagnosis of hypertension
RAH: Hypertensive with a diagnosis of kidney disease
VIP: Variable importance in projection
PLS-DA: Partial least squares-discriminant analysis
DBP: Diastolic blood pressure
SERS: Surface-enhanced Raman spectroscopy
KD: Kidney disease
CVD: Cardiovascular disease
DM: Diabetes mellitus
AuNPs: Gold nanoparticles
Cal: Calibration
CV: Cross validation
Pred: Prediction
ROC: Receiver operating characteristic curve
DLS: Dynamic light scattering
2ndD: Second derivative
LV: Latent variables
SenCal: Sensitivity calibration
SenCV: Sensitivity cross validation
SenPred: Sensitivity prediction
SpeCV: Specificity cross validation
SpePred: Specificity prediction
AccCal: Accuracy calibration
AccCal: Accuracy calibration
AccPred: Accuracy prediction
