# Supplementary material for: Surface-enhanced Raman Spectroscopy in urinalysis of hypertension patients with kidney disease
Source: Sci Rep. 2024 Feb 6;14:3035. doi: 10.1038/s41598-024-53679-9 (PMC10847430; doi:10.1038/s41598-024-53679-9)
Supplement: Supplementary file 1 — Supplementary Information 1. [file 41598_2024_53679_MOESM1_ESM.docx]

Supplementary Material

1. **Patient distribution**

In this research, the donors' genders for the model development correspond to the female and male genders, which are distributed in 3 races: Indigenous (Ind), Afro-Colombian(Afro), and Mestizo (Mez).

The donors for the study are distributed in the two classes see the table.

Table A. Patient distribution in gender and race

|  | Race | Female | Male |
| --- | --- | --- | --- |
| HV | Ind | 6 | 4 |
|  | Afro | 8 | 7 |
|  | Mez | 36 | 14 |
| AH | Ind | 3 | 8 |
|  | Afro | 10 | 18 |
|  | Mes | 17 | 31 |
|  | Total | 80 | 82 |

Considering the different factors that are linked to each race and gender, which generates the probability that the relationship of biomarkers present in the different urine samples will be modified, as the object of study is two specific hypertensive and non-hypertensive populations, it is expected that the relationship of biomarkers is different than the proposed method has the ability to differentiate. By having all this variability, we hope the model will be more robust, allowing its differentiation capacity to be more precise.

Based on the results obtained in the development of the model, the variability corresponding to gender and race of the different samples did not significantly impact the differentiation capacity between AH and HV. This is because there is a homogeneous distribution between gender, race, and class.

1. Machine learning comparison model

Several machine learning methods were tested, such as Logistic Regression, SVM, Random Forest, KNN, Decision Tree, Gradient Boosting, MLP, AdaBoost, Gaussian Naive Bayes, and Ridge Classification. We found that there was no significant improvement in the results.

Table B.


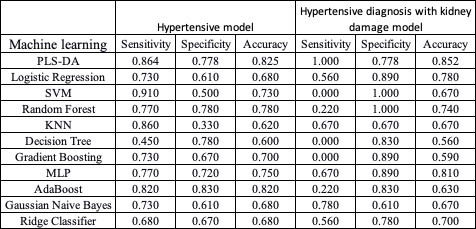


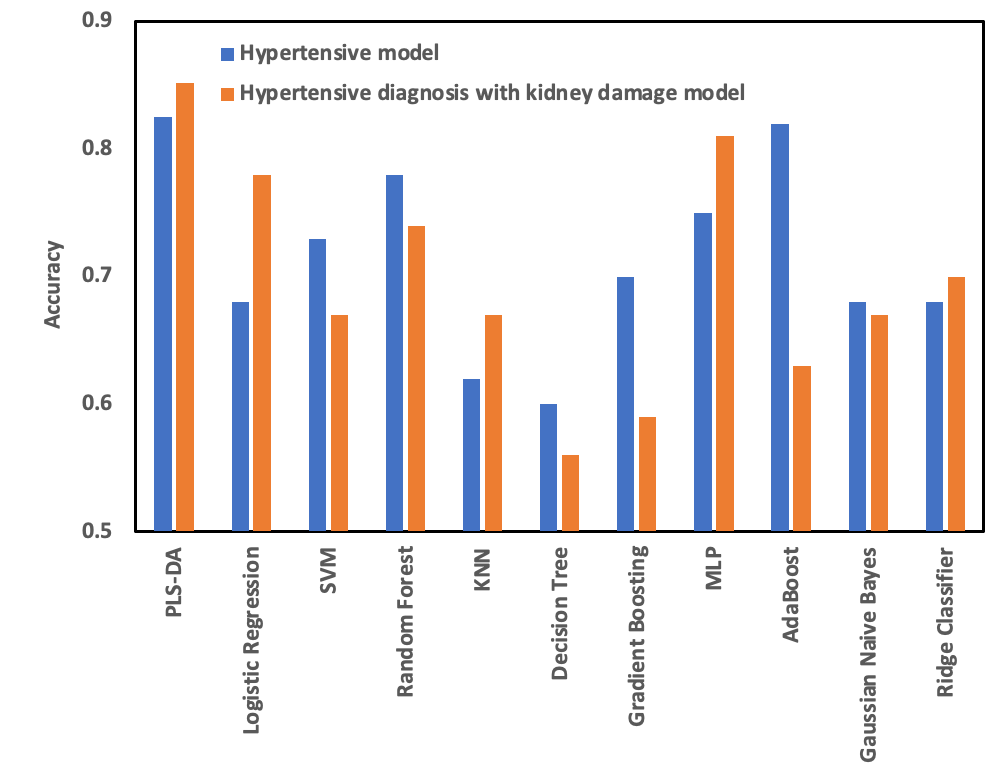


**Figure A. Comparison of accuracy for the different Machine Learning methods**
